# Colored cotton crop wastes valorization through pyrolysis: a study of energetic characterization and analytical Py-GC/MS

**DOI:** 10.1038/s41598-024-60019-4

**Published:** 2024-04-23

**Authors:** Janduir E. Silva, Joemil O. Deus Junior, Guilherme Q. Calixto, Dulce M. A. Melo, Marcus A. F. Melo, Vital C. B. Júnior, Bruna M. E. Chagas, Everaldo P. Medeiros, Renata M. Braga

**Affiliations:** 1 Centro de Tecnologia, Programa de Pós-graduação em Engenharia Química, Universidade Federal do Rio Grande do Norte, Natal, RN CEP: 59078-970 Brazil; 2 Centro de Tecnologia, Dep Eng. Química, Universidade Federal do Rio Grande do Norte, Natal, RN CEP: 59078-970 Brazil; 3 Centro de Ciências Exatas e da Terra, Instituto de Química, Universidade Federal do Rio Grande do Norte, Natal, RN CEP: 59078-970 Brazil; 4 Escola Agrícola de Jundiaí, Universidade Federal do Rio Grande do Norte, Macaíba, RN CEP: 59280-000 Brazil; 5 Empresa Brasileira de Pesquisa Agropecuária (EMBRAPA), Campina Grande, PB CEP: 58428-095 Brazil

**Keywords:** Environmental sciences, Chemistry

## Abstract

The present work aimed to study different parts of colored cotton waste through energetic characterization and analytical flash pyrolysis. Stalks and bolls of BRS cotton cultivars from Sementes do Brasil (Green, Ruby, Topaz and Jade) were studied, using white cotton (BRS 286) as a comparison. The energetic potential of biomass was evaluated by bulk density, High Heating Value (HHV), proximate and ultimate analysis, compositional and thermogravimetric analysis (TGA). Pyrolysis was performed in a micro-pyrolyzer and the products were identified by gas chromatography and mass spectroscopy (Py-GC/MS). The results indicated a significant energetic potential, suggesting that can be used as an alternative energy source for thermochemical processes. The results of conventional pyrolysis indicated the presence of oxygenated compounds of different organic groups: aldehydes, ketones, phenols, furans and ethers, characteristic of the decomposition of lignocellulosic materials. Light organic acids in the C1-C4 range stood out the most, followed by phenols that appeared in a considerable proportion. Finally, it is concluded that the energy potential and pyrolysis products of the different parts (stalks and bolls) of colored cotton waste can be used to generate bioenergy and various chemical compounds of plant origin from green chemistry.

## Introduction

Cotton (*Gossypieae*) is a versatile plant of tropical origin which has easy adaptation to soil and climate conditions, and is capable of presenting high resistance to drought, thereby being a cultivar of great relevance for the semi-arid regions of the planet. These factors contribute to the development of cotton plantations, constituting an activity of great economic and social relevance. Cotton is the most consumed natural fiber in the textile industry.

Brazil is currently a major cotton producer. A total of 2.8154 thousand tons were produced in 2021, a volume capable of meeting the country’s domestic demand and exporting the surplus. Consequently, large amounts of cotton waste are produced. The production chain is geared towards textile, food, cosmetics and biofuels in the form of cottonseed oil. The greatest market demand is met by white fiber, but naturally colored fibers have been gaining ground and generating new business opportunities for those who prefer organic or agroecological management^1^.

Commercial colored cultivars were developed by EMBRAPA (Empresa Brasileira de Pesquisa Agropecuária). The first cultivar, BRS 200 Brown, was registered in the RNC in 2001. Then, BRS Green, BRS Ruby and BRS Sapphire were registered in 2004, BRS Topaz in 2010, and BRS Jade in 2017, all with *G. hirsutum* var.hirsutum germplasm^2,3^.

These cultivars can be planted in areas zoned for arboreal cotton cultivation, and can also be explored under irrigation. The characteristics of the species combined with its higher added value (when compared to white cotton) has contributed to attracting the interest of farmers, and has consequently increased the number of plantings of this cotton in the Northeast region. Combined with the production of cotton fibers, a large amount of agricultural waste is generated. It is estimated that the amount of cotton crop residues generated annually in Brazil alone is approximately 0.045 tons per hectare^4^. The use of colored cotton waste for energy generation can add economic value to the industrial process, as well as contribute to environmental sustainability, avoiding waste and promoting a production chain that does not harm the environment.

The high demand for energy has stimulated research involving alternative energy sources. Cotton agricultural residues have great potential for distributed energy generation as showed by Silva^5^, which compared briquettes obtained from cotton wastes and discovered that colored cultivars generate briquettes with good mechanical and physicochemical characteristics, being suitable for energy generation via biomass densification. However, this biomass can also be used to generate energy through the production of biofuels. Barisçi and Oncel^6^ studied the pyrolysis of combed cotton waste in a fixed bed reactor. The bio-oil obtained had a heating value of 11.49 MJ/kg and several chemical compounds in its composition, including furans, ketones, aldehydes, carboxylic acids and hydrocarbons. They showed that the use of cotton waste can be a viable alternative for energy production. Krishna et al.^7^ observed that the cotton waste had an elemental composition of 44.90% C, 5.25% H, 0.83% N and 0.27% S. The volatile and ash content was 90% and 4.75%, respectively. Moreover, 51.40% of C, 4.00% of H, 1.33% of N and 43.24% of O were obtained in the elemental analysis. These results indicate the great potential of using this residue via thermal conversion. Madhu et al.^8^, and Ali et al.^9^ also showed satisfactory results regarding bio-oil production from white cotton crop residues. However, there are few studies on the energy potential of colored cotton cultivars in the literature.

Based on the large amount of cotton waste generated in Brazil due to high productivity, with the prospect of a gradual increase in the production of colored cotton which has been conquering an increasingly demanding market in terms of organic or agroecological management, the objective of this study was to perform an energetic characterization and evaluation of the products of conventional pyrolysis of stalks, bolls and the mixture of these parts of the colored cotton, aiming to promote the energetic use of this biomass and/or the conversion of the pyrolysis products into chemical products of industrial interest, contributing to diversify the energy matrix, reducing possible environmental impacts caused by the disposal of this waste.

## Experimental section

**Figure 1:**
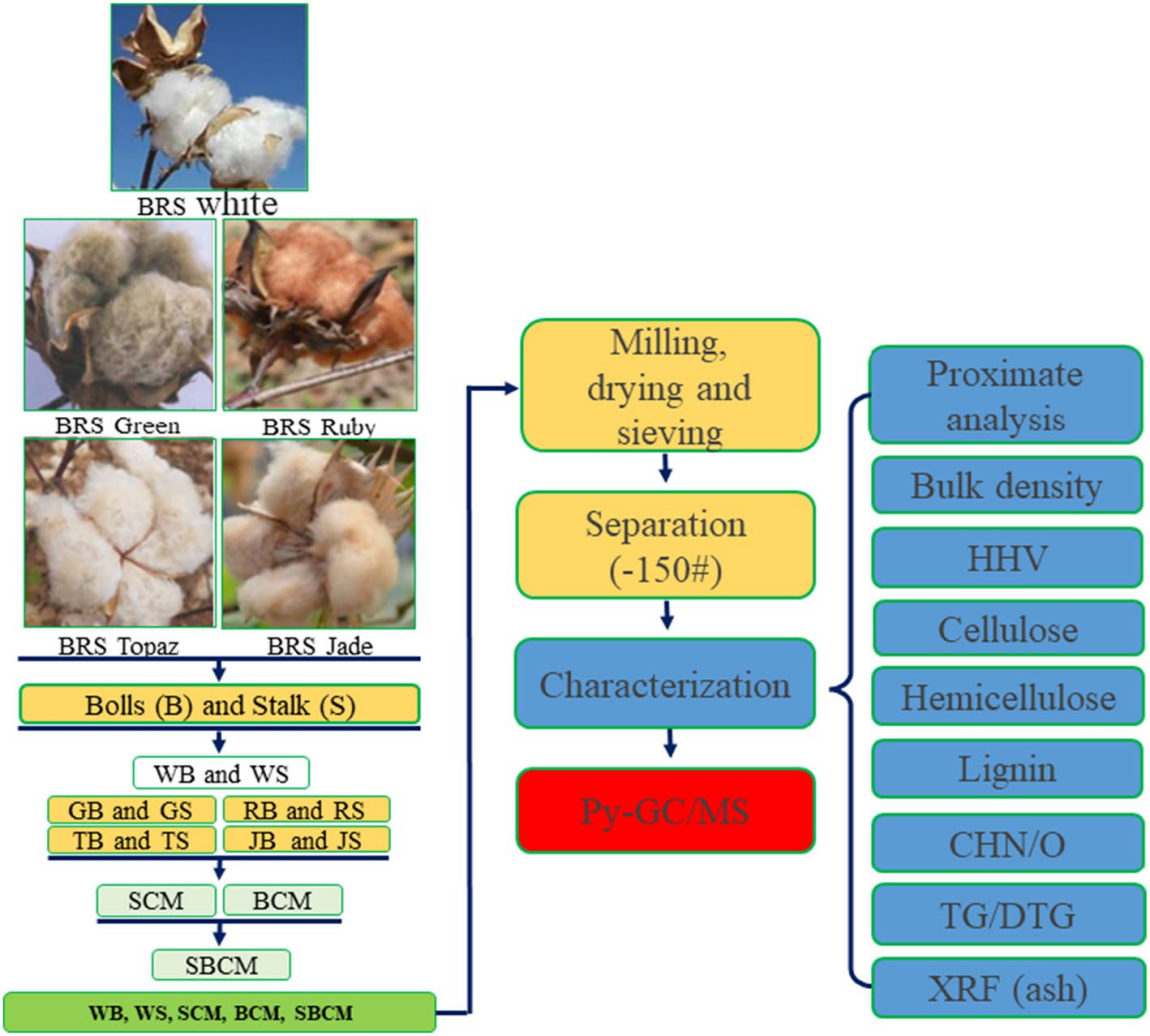
Methodological schedule.

Biomass from the cultivation residues of Brasil Sementes—BRS Green, BRS Ruby, BRS Topaz and BRS Jade were studied, which were collected at a farm belonging to the Empresa de Pesquisa Agropecuária do Rio Grande do Norte (EMPARN), located in the municipality of Apodi, RN, Brazil. The region has a very hot and semi-arid climate according to the Köppen climate classification, with an average annual temperature of 27.1 °C, an average maximum temperature of 34.1 °C and an average minimum temperature of 22.8 °C. The area is classified as having Cambisol soil with a loamy loam texture. A summary of the methodology used in this research is presented in Fig. 1.

The collection of cultivation residues took place after the cotton harvest. Then, this material was separated into stalks (S) and bolls (B), which were dried in the sun and crushed in forage, separately, obtaining different granulometric ranges. These biomasses were sieved and granulometry between 0.104 and 0.074 mm was selected.

Next, 13 samples were used, 5 from bolls, 5 from stalks and 3 mixtures, which were named according to the part of the cotton plant and the color of its fiber, thus, the names of the samples were formed by the initial letter B or S, bolls and stalks, respectively, followed by a letter representing the fiber color of that cotton used, with WB, GB, RB, JB and TB referring to the white, green, ruby, jade and topaz cotton boll samples, respectively, and WS, GS, RS, JS and TS referring to the stalk samples of these same species. The sample called BCM was formed by mixing the boll samples in equal proportions (1:1:1:1); and the sample called SCM resulting from the mixture between the stalk samples (1:1:1:1), and a BSCM sample resulting from the mixture between the BCM and SCM samples (1:1).

The energetic characteristics of the biomasses selected in this study were evaluated through the bulk density, calculated according to the ASTM E 873-82 proximate analysis, which consists of determining the moisture content (E 871-82), volatiles (E 872 and E 897), ash (E 1755-01) and fixed carbon by difference. Higher heating value was determined using a Parr bomb calorimeter following ASTM E711-87. Ultimate chemical analysis (CHN/O) was performed on an Elementary Series II CHNS/O Analyzer PerkinElmer 2400. The oxygen content was calculated by the difference (%O = 100% − %C − %H − %N − %H_2_O). The evaluation of the thermal behavior of the biomass samples was performed on a TA Instruments SDT Q600 thermogravimetric scale. This analysis was performed from room temperature to 900 °C with a heating rate of 10 °C min^−1^, under a flow of 100 mL min^−1^ of N_2_, and using approximately 10 mg of the samples.

The cellulose and hemicellulose content were determined by the methodology developed by Van Soest and Wine^10^ using the Acid Detergent Fiber (ADF) and Neutral Detergent Fiber (NDF) assays; the lignin content was determined by acid hydrolysis using the Klason 1893 method.

The constituent metals of biomass ash were determined by energy dispersive X-ray fluorescence (XRF) using a Shimadzu EDX-820 device. The X-ray fluorescence spectra were obtained using about 300 mg of biomass in powder form with a particle diameter between 0.104 and 0.074 mm, deposited in a polyethylene sample holder.

Analytical pyrolysis was performed at 500 °C using a CDS Analytical 5200 HP-R micro pyrolyzer connected to a gas chromatograph (3800 VARIAN) with mass spectrometry detection (GC/MS). Approximately 1 mg of the biomass was trapped in a small quartz tube by inserting glass wool at the ends of the tube, and then heated rapidly to 500 °C by a platinum filament around this tube. The vapors from the thermal decomposition of the biomass were drawn through the N_2_ flow of 50 mL min^−1^, stored in a Tenax trap and desorbed at 300 °C for 4 min, then injected through split mode 1:50 on a VARIAN 3900 chromatograph with a VF-5MS chromatographic column (30 m × 0.25 mm × 0.25 µm). The column heating temperature program was adjusted to start the analysis at 40 °C, remaining at this temperature for 2 min, then heated at 10 °C min^−1^ to 280 °C, and remaining for 14.5 min. Pyrolysis products were identified through spectral similarity greater than 85% using commercial Wiley NBS and NIST mass spectra libraries. The semi-quantitative approach was based on the internal normalization method, considering only the areas/mass (area/mg) of the identifiable peaks of each organic compound.

The results of the statistical analyzes were submitted to descriptive statistics to understand them with greater reliability. Standard deviations, median and mean difference within the dispersion of the sample were calculated (t-test, 95% confidence). Confidence intervals were calculated from the analysis function in “compare means: One-sample t-test”, in the IBM SPSS Statistics version 22, trial license software program.

## Results and discussion

### Biomass characterization

**Figure 2:**
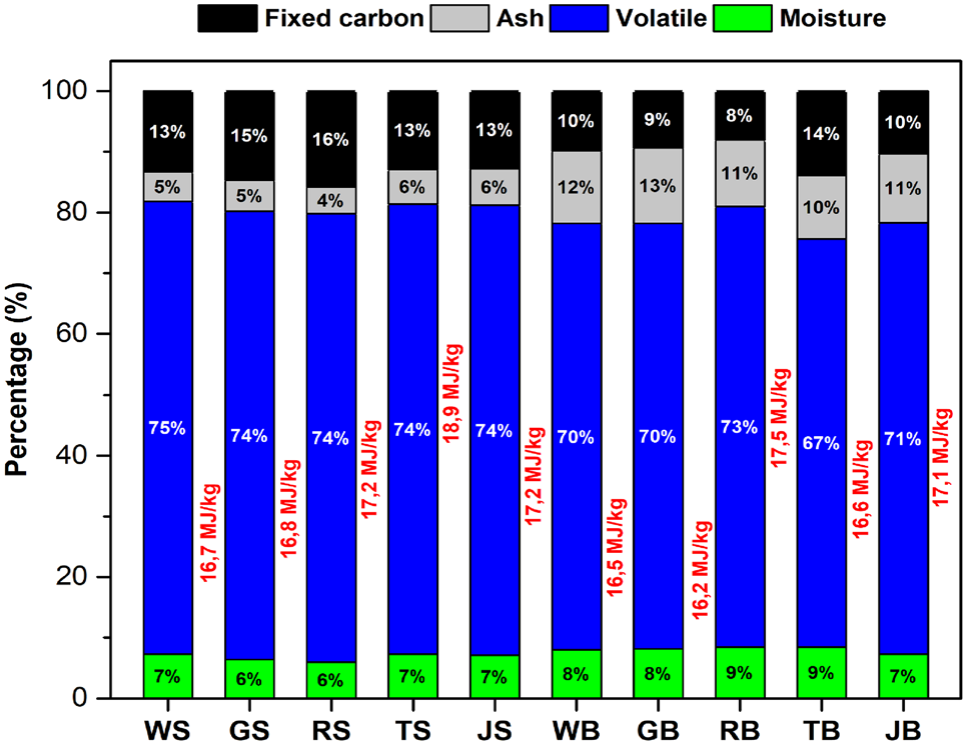
Proximate analysis and higher heating value (HHV) of SB, SG, SR, ST, SJ, WB, GB, RB, TB and JB samples.

The proximate analysis and the heating value of the parts (stalks and bolls) of each cultivar are shown in Fig. 2.

All analyzes were performed in triplicate, with the mean of each variable and the respective standard deviation, which varied from 0.06 to 0.4. According to the results, all biomasses presented volatile contents ranging from 67 to 75% and moisture lower than 10%. The stalks generally showed lower ash and fixed carbon contents than the bolls. The higher ash contents may be related to the greater capacity of the boll structure to accumulate mineral material brought by the wind. The heating value ranged from 16.2 to 18.9 MJ/kg, emphasizing the potential of these residues for energy applications via thermal conversion for bio-oil production. These results are in agreement with works found in the literature on white cotton (Guo et al.^11^, Kataria et al.^12^, Sui et al.^13^) and also for other lignocellulosic biomasses (Hossain et al.^14^, Qiu et al.^15^, Sellin et al.^16^). S1 Table presents the mean and median of all measurements of moisture, volatiles, ash, fixed carbon and heating value of the samples.

According to S1 Table, the mean ​moisture and volatile values are within the CIs confidence intervals [6.85; 8.06] and [70.35; 73.86], respectively, showing that these samples presented similar moisture contents. The same can be observed with volatile content, indicating that the bio-oil produced will have a good yield of approximately 67% for bolls and 75% for stalks. Moisture values ​of less than 10% indicate that the fuel produced is likely to have good ignition and therefore better quality.

The stalks had ash content values ​below (up to 4.4%) the lower limit of the CI and the bolls were above (up to 12.5%) the upper limit of the CI. It is noted that only 3 samples had fixed carbon values ​below (up to 8.0) the lower limit of the CI, with these being the stalk samples. Finally, it is observed that only two boll samples (WB and GB) had their higher heating values ​below the lower limit of the CI [16.53; 17.60]. This can be explained by the high ash content of these samples (Fig. 2). Higher volatile and fixed carbon contents promote high heating value since they consist of chemical energy stored in the biomass^17^.

Regardless of the cotton cultivars, their energetic properties are very similar, with the biggest difference being observed between the different parts (stalks and bolls), whether of the same species or not. The prediction is that the mixture of all the stalks or all the bolls of the different species present results close to the values ​obtained individually. It is also interesting to study the energetic potential of the mixture of stalks and bolls of all colored species to verify if there is a need to separate them.

**Figure 3:**
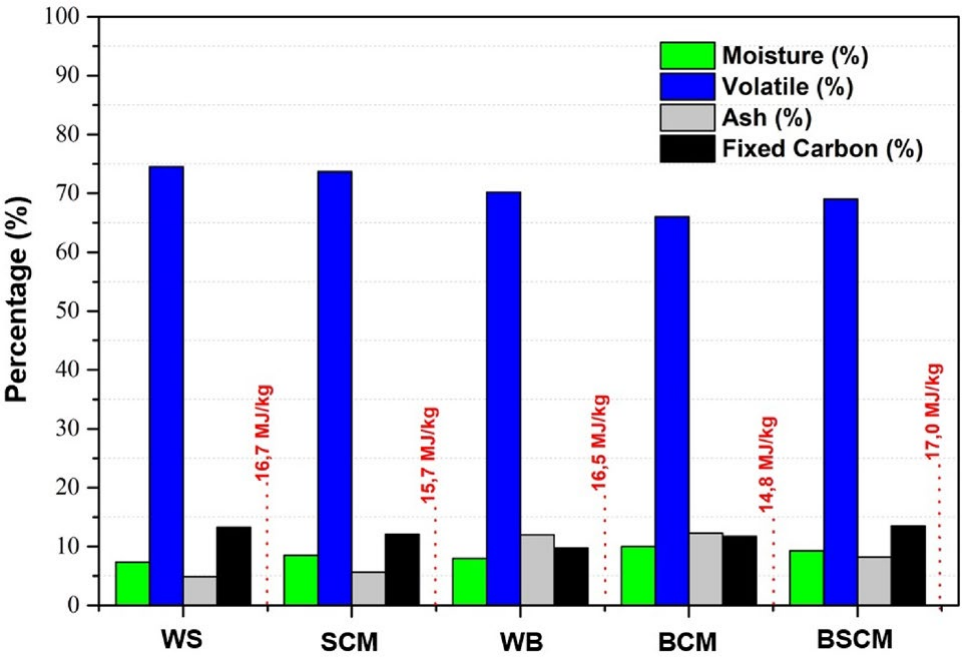
Proximate analysis and higher heating value (HHV) of WS, SCM, WB, BCM and BSCM samples.

The mean proximate analysis and heating values of WB, WS, BCM, SCM and BSCM samples, obtained in triplicate and all with standard deviations ranging between 0.02 and 0.4, can be seen in Fig. 3.

The values​referring to descriptive statistics: mean, median and standard deviations, as well as the confidence interval for each variable (t-test for means of a sample), obtained in accordance with Fig. 3, are shown in S2 Table.

According to S2 Table, it can be seen that the values​referring to the proximate analyzes and higher heating value of the colored cotton biomass are similar to those observed in S1 Table. It appears that BCM and WS had the highest and lowest content regarding moisture (10% and 7.32%), respectively, as shown in Fig. 3, which are very close to the upper and lower CIs.

It also appears that the BW, BCM and BSCM samples presented volatile content values​close to the lower CI [66.34], while the opposite occurs in relation to WS and SCM samples, which have values​close to the upper CI [75.01] (S2 Table). The ash contents of all samples had values​within the confidence interval.

The CIs [10.23; 13.92] for fixed carbon show that only the WB sample presented a value below (up to 9.8) the lower confidence limit, and that the WS, SCM and BSCM samples had their values​close to the upper CI. Finally, the CI [15.03; 17.24] for the Higher Heating values shows that only the BCM sample presented a value below the lower CI. This can be explained by its low volatile content, high ash and moisture content (Fig. 3) when compared to the other samples.

It is known that for an energy application process, the volatile material and fixed carbon content of the biomass used is important, since high and low levels of volatile material and fixed carbon, respectively, favor rapid burning of the fuel. It is observed that the colored cotton waste evaluated in this study, as well as their mixtures, presented high values of volatile material and low values of fixed carbon, ash and moisture, which makes these biomasses attractive for the production of biofuels, since high levels of moisture and ash decrease the calorific value. Ash significantly reduces the heating value of biomass since it acts as a catalyst in fast pyrolysis, promoting the formation of gas and char, at the expense of bio-oil.

The bulk density and its relation with HHV for the WS, WB, SCM, BCM and BSCM samples described in Table 1 are indicative to evaluate the capacity of energy generation by combustion.

**Table 1:** Bulk density and energy density of the samples.

Biomass	Bulk density (kg/m^3^)	Energetic density (MJ/m^3^)
WB	320	5.344
WS	300	4.950
BCM	363	5.699
SCM	380	5.624
BSCM	350	5.950

It is possible to observe that samples from colored cotton release more energy during combustion when compared to white cotton, as they have higher bulk and energy density, especially the BSCM sample. Among the colored cotton samples, it is noted that those with higher apparent densities (BCM and SCM) have low energy densities in relation to BSCM (lower bulk density). This may be related to the different morphologies and compositions of the lignocellulosic material, such as: cellulose, hemicellulose and lignin (Table 2), carbon, hydrogen, nitrogen and oxygen (Table 3) and mineral extracts (Table 4); however, it is evident that mixing the different parts of the colored cotton in equal proportions is more advantageous for energy generation.

**Table 2:** Cellulose, hemicellulose and lignin contents.

Analysis	Samples				
	WB	SCMWS	BCM	SCM	BSCM
Cellulose (%)	38.8 ± 0.05	38.9 ± 0.04	37.9 ± 0.02	33.5 ± 0.03	38.2 ± 0.04
Hemicellulose (%)	21.0 ± 0.06	24.0 ± 0.03	27.1 ± 0.07	25.7 ± 0.05	25.8 ± 0.05
Lignin (%)	16.5 ± 0.07	16.1 ± 0.07	13.4 ± 0.05	14.2 ± 0.06	14.7 ± 0.02

**Table 3:** CHN/O analysis results.

	Carbon (%)	Hidrogen (%)	Oxygen (%)	Nitrogen (%)	H/C	O/C	Empirical formula
WB	42.7 ± 0.04	6.4 ± 0.06	50.2 ± 0.07	0.71 ± 0.05	0.15	1.17	CH_1.8_O_0.88_N_0.014_
WS	40.5 ± 0.03	6.07 ± 0.05	52.6 ± 0.06	0.77 ± 0.03	0.15	1.30	CH_1.8_O_0.98_N_0.016_
BCM	38.9 ± 0.05	6.0 ± 0.04	54.0 ± 0.05	1.09 ± 0.04	0.15	1.40	CH_1.85_O_1.04_N_0.024_
SCM	44.26 ± 0.04	6.75 ± 0.07	47.2 ± 0.06	1.8 ± 0.05	0.15	1.10	CH_1.83_O_0.8_N_0.035_
BSCM	41.1 ± 0.07	6.4 ± 0.05	51.0 ± 0.03	1.62 ± 0.04	0.16	1.24	CH_1.86_O_0.93_N_0.034_

**Table 4:** Ash composition, determined by X-ray fluorescence (XRF).

Component	WB (%)	WS (%)	BCM (%)	SCM (%)	BSCM (%)
K_2_O	48.4	57.0	57.0	49.6	53.4
CaO	37.4	28.0	28.4	34.0	32.5
SO_3_	4.0	6.0	6.0	5.3	5.6
MgO	3.7	2.0	–	3.5	1.6
P_2_O_5_	3.5	2.6	0.9	2.6	1.3
Al_2_O_3_	1.2	–	–	1.0	–
SiO_2_	0.7	2.0	2.3	1.4	1.6
Fe_2_O_3_	0.7	1.8	4.0	1.5	2.6
Others	0.4	0.6	1.4	1.1	1.4

It is possible to infer that the cellulose and lignin contents (Table 2) are higher in the stalk samples. However, the hemicellulose content is slightly higher in the boll samples; in addition, there is not a considerable difference in the values in any of the cases. The composition of these constituents in the BSCM sample were intermediate when compared to the values​observed for stalks and bolls, as expected. Cellulose and hemicellulose contribute to a higher yield of bio-oil in pyrolysis, whereas the higher the lignin content, the higher the charcoal yield^18^.

The chemical formulas of each biomass were calculated from the CHN/O contents (Table 3), considering the molar ratio of each chemical element with carbon. It is observed that the amount of hydrogen in the samples has the following trend: WS = WB < SCM < BCM < BSCM, highlighting SCM presented the highest amount of hydrogen, H/C ratio, heating value (Fig. 3) and energy density (Table 1).

The elemental contents of C, H, N, S and O are important to determine the HHV of biomass. The high values of C and H and low values of O have the potential to increase the HHV, favoring the production of quality biofuel. According to the chemical formulas, the BCM sample presented nitrogen contents and energy density lower than the values​observed for SCM, due to the fact that this biomass has a greater amount of oxygen and a greater O/C ratio^19^.

The ash composition in the biomass depends on the species and plant part, as well as the nutrients available during cultivation, soil quality, fertilizers and climatic conditions, as they are important factors for determining the potassium, sodium, chlorine and phosphorus contents in the biomass ash^20^. The high potassium content in the samples is due to the fertilization process, which consists of adding fertilizers such as NPK (nitrogen, phosphorus and potassium) to the soil.

Studies indicate that the presence of metals and their oxides in the ash content of biomass can have a catalytic effect on the pyrolysis process, working with active sites capable of increasing the surface area^21^. Wang et al.^22^ demonstrated that CaO, MgO and Al_2_O_3_ oxides are able to improve the quality of final products and the CaO used as catalysts during pyrolysis of biomass could reduce the CO and CO_2_ content through reactions of carbonation and dehydration. According to Nassar et al.^23^ pyrolysis of biomass with metal oxides may produce products with more stability, since they decrease the viscosity and oxygenated products content.

According to Table 4 results, it is observed that the ash of all evaluated biomasses showed high potassium and calcium levels. The third most predominant element in the biomasses was P. These results are expected, since agro-industrial residues have high levels of K, Ca and P. It is verified that the ash content of the stalks (WS and SCM) presented similar values. The same was observed for the bolls (WB and BCM). Higher Ca and P contents and the presence of Al were generally observed in the stalks when compared to the bolls which presented higher K, Si and Fe contents, but did not present Al in their constitution. In addition to these constituents, there was also the presence of trace elements such as: Mn, Br, Ti, Zn, Ni, Cu and Cr, which were counted as *others* in Table 4. The ash has a catalytic effect in the pyrolysis process, where the metals can affect the yield of pyrolysis products, reducing the amount of condensable liquids and increasing coal production, in addition to promoting depolymerization reactions for the formation of oxygenated molecules of lower molecular weight (Carpenter et al.^24^, Morin et al.^25^). For example, the presence of K in the biomass has a catalytic effect during pyrolysis, promoting the formation of phenols in the bio-oil^26^. Case et al.^27^ evaluated the influence of calcium present in the biomass during the pyrolysis process, having observed that the presence of this metal disfavors the formation of levoglucosan and favors the formation of alkylated phenols, polyaromatic hydrocarbons and alkyl-substituted cyclopentenones.

**Figure 4:**
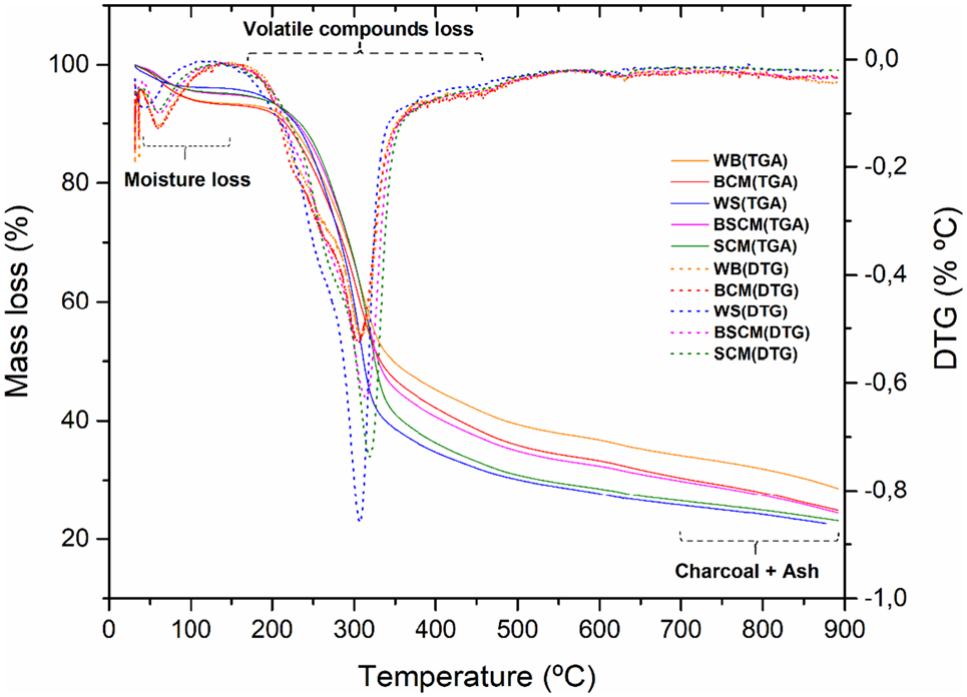
TG/DTG thermal analysis curves of WB, WS, BCM, SCM and BSCM samples.

The results of the thermogravimetric analysis (TGA) of the studied biomasses show three main mass loss events (Fig. 4). Similar mass losses in the first event regarding moisture release were observed for WB and BCM samples (6.7 and 7.0%), as well as WS and SCM samples (4.5 and 4.6%). These values are similar to those obtained in the proximate analysis (Fig. 3), which can also be observed in relation to the BSCM sample which has an intermediate value between the SCM and BCM samples.

The second stage is characteristic of hemicellulose and cellulose decomposition, being responsible for most of the volatile content, in which the stalk samples, especially SCM and WS which showed the highest mass losses, ∆m = 58.8% and ∆m = 59.5%, respectively. These results followed the same trend observed in Fig. 3 regarding volatile content. The last step occurs in a wide temperature range from 250 °C to around 900 °C, indicating lignin degradation. The different thermal behavior observed in each sample can be attributed to the contents of each component and also to the influence of the different metals present in each biomass, which have catalytic effects. Thermal decomposition of hemicellulose and cellulose occurs at 220 to 315 °C and 315 to 400 °C, respectively^28^.

It was also observed that the ash and charcoal contents of the boll samples were higher at 700 °C than those from the stalks, following the following order: WB > BCM > BSCM > SCM > WS. A similar result can be observed in relation to the sum of the ash content and percentage of fixed carbon (Fig. 3), obtained from the immediate analysis, whose relationship is BCM > WB > BSCM > WS > SCM. The same trend can be observed in relation to the presence of K in the ash composition of these biomasses (Table 4). Kelkar et al.^29^ observed that a greater amount of alkali metals, such as potassium in the biomass, promotes increased coal and gas yield, reducing the amount of bio-oil.

### Pyrolysis

**Figure 5:**
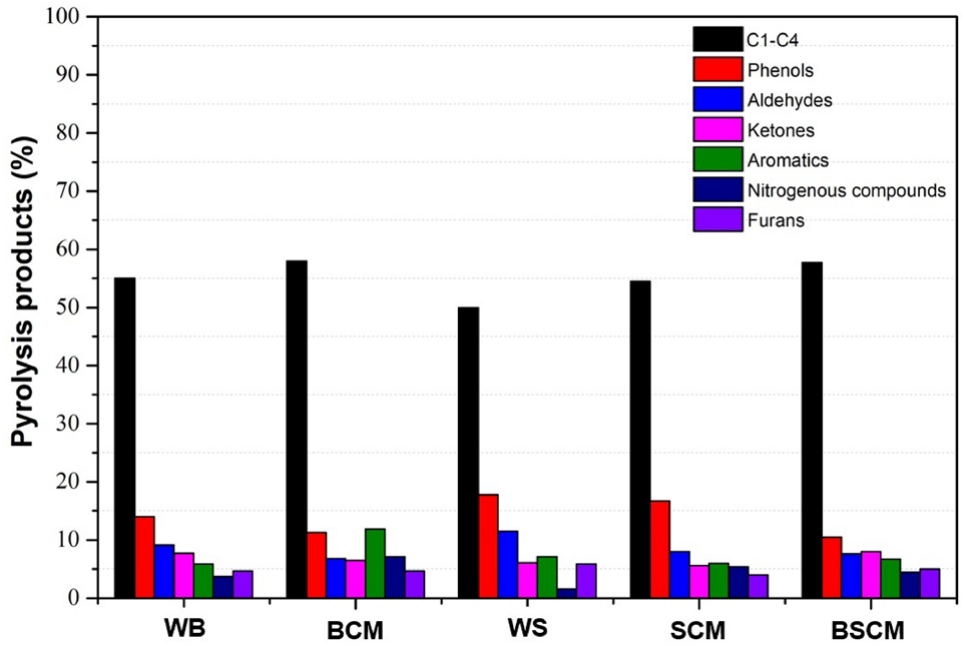
Main products of conventional pyrolysis.

The conventional pyrolysis biomass products are shown in Table 5 and Fig. 5. It is possible to observe the presence of oxygenated compounds of different organic groups: aldehydes, ketones, phenols, furans and ethers, which are characteristic of the decomposition of lignocellulosic materials, as described in the literature (Braga et al.^30^; Calixto et al.^31^).

**Table 5:** Products of the conventional pyrolysis of biomass.

RT (min)	Compound	Group	WB_Py	BCM_Py	WS_Py	SCM_Py	BSCM_Py
1.56–4.11	NI	C1–C4	∑ = 54.6	∑ = 52.85	∑ = 49.9	∑ = 54.52	∑ = 57.70
4.70	2.5-Dimethyl-furan (C_6_H_8_O)	Furan	0.97	1.29	1.74	1.00	1.26
5.30	1-Methyl-1 H-pyrrole (C_5_H_7_N)	Nitrogenated	2.73	4.63	0.81	3.09	3.35
5.60	Pyrrole (C_4_H_5_N)	Nitrogenated	1.43	1.83	0.81	2.28	1.19
5.88	Toluene (C_7_H_8_)	Aromatic	3.61	7.21	4.42	3.92	4.61
6.34	Cyclopentanone (C_5_H_8_O)	Ketone	1.09	1.94	0.87	0.77	1.26
7.25	3-Furaldehyde (C_5_H_4_O_2_)	Aldehyde	9.08	6.24	11.51	7.98	7.58
8.11	p-Xylene (C_8_H_10_)	Aromatic	1.81	4.31	1.93	1.32	2.08
8.89	2-Methyl-cyclopenten-1-one (C_6_H_8_O)	Ketone	3.28	3.01	2.61	2.35	3.72
8.90	2-Ethyl-5-methylfuran (C_7_H_10_O)	Furan	3.70	3.01	4.17	2.89	3.72
10.39	Phenol (C_6_H_6_O)	Phenol	3.53	2.91	3.86	3.25	3.35
11.78	2.3-Dimethyl-cyclopenten-1-one (C_7_H_10_O)	Ketone	2.10	1.83	1.62	1.61	1.93
11.98	o-methyl-phenol (C_7_H_8_O)	Phenol	1.72	1.72	2.18	1.83	1.71
12.38	p-methyl-phenol (C_7_H_8_O)	Phenol	2.23	2.15	2.6	2.41	1.78
12.83	o-Methoxy-phenol (C_7_H_8_O_2_)	Ether/phenol	4.16	2.15	5.48	5.92	1.93
13.10	4.4-Dimethyl-cyclohexen-1-one (C_8_H_2_O)	Ketone	1.18	1.08	1.00	0.90	1.12
13.89	2.3-DimethylPhenol (C_8_H_10_O)	Phenol	1.01	0.54	1.24	1.13	0.89
14.60	o-Methoxy-m-methyl-phenol (C_8_H_10_O_2_)	Ether/PHENOL	0.46	0.32	0.81	0.58	0.37
14.86	p-Methoxy-m-methyl-phenol (C_8_H_10_O_2_)	Ether/phenol	0.76	0.54	1.43	1.61	0.45
15.64	3.4-Dimethyl-toluene (C_9_H_12_O_2_)	Ether/aromatic	0.50	0.43	0.75	0.64	NI

*RT* retention time (min), *NI* not identified.

Lignocellulosic biomass has a complex structure mainly consisting of cellulose, hemicellulose, lignin, extractives and inorganic material. A postulated mechanism for the conversion of cotton waste by the fast pyrolysis process at 500 ºC was proposed in this study (Fig. 5). The pyrolysis process occurs through primary and secondary reactions. The chemical bonds of the polymers are broken in heating the biomass, releasing volatile compounds which react to form the pyrolysis products. Some of the compounds produced are unstable and react through secondary cracking reactions^32^. Thus, depolymerization and fragmentation occur as the main primary mechanism steps to form bio-oil. The bio-oil composition is generally related to the structures of the biomass compounds and the pyrolysis parameters.

**Figure 6:**
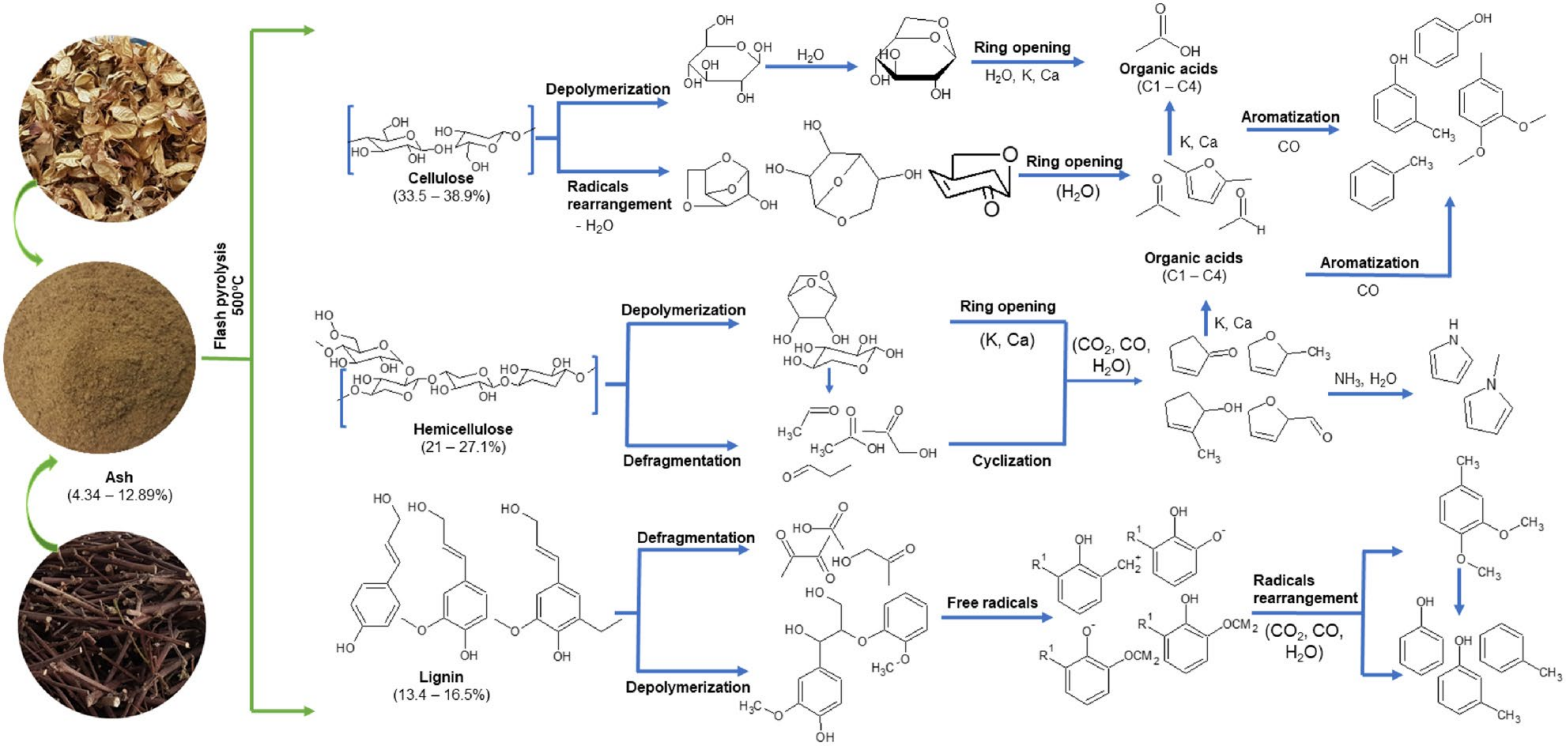
Postulated mechanism.

The thermal degradation of the cellulose chains mainly promotes the formation of glucose, and levoglucosan is formed in a second stage after the elimination of a water molecule, followed by the formation of furans, ketones, acids, aldehydes, alcohols, phenols and hydrocarbons^33^. Hemicellulose is the least stable component of the lignocellulosic biomass structure, basically being formed by hexoses and pentoses that are easily decomposed by heat treatment via decarbonylation and oligomerization reactions, thereby originating acids, ketones, furans, phenols, alcohols and hydrocarbons^34^. Phenolic aromatic compounds are typically formed from the macromolecular structure of lignin, as lignin is composed of phenylpropane structural units in a three-dimensional framework. Thus, the main components of lignin bio-oil are phenols^35^. The main compounds arising from the thermal degradation of this biomass can be seen in Fig. 6.

Aromatic hydrocarbons, such as toluene and p-xylene, were identified in the pyrolysis vapors of colored cotton bolls and stalks, which makes these residues attractive as a source of raw material for the production of quality bio-oil. The highest yields of toluene and p-xylene were observed for the BCM sample—7.21 and 4.31%, respectively. Nitrogen products were produced, but oxygenated compounds were predominant in the pyrolysis of all samples.

The nitrogen compounds, 1-Methyl-1H-pyrrole and pyrrole were formed by the pyrolysis of cotton waste for all evaluated conditions and the lowest percentages were observed for the WS sample. According to the postulated mechanism, pyrrole is formed from the conversion of furan by the ammonization process^36^, which occurs as a result of the thermal degradation of the protein content present in the samples.

Organic acids containing one to four carbons in the chain (C1-C4), such as acetic acid and methyl acetate stood out among the classes of compounds present in the pyrolysis products of the analyzed biomasses, in which the percent ranged from 49.9 to 57.7% (Table 5). This result may be associated with the ash content of the samples and its catalytic effect. Chemical compounds produced during primary pyrolysis reactions tend to react through secondary cracking and condensation reactions due to the presence of inorganic metals, which results in changes in the chemical distributions in the bio-oil and its yield. Levoglucosan is formed by the reactions of breaking and rearrangement of chemical bonds of cellulose molecules in the range of 400–550 ºC, being one of the predominant components of the bio-oil of lignocellulosic biomasses^37^. The presence of mineral elements in the biomass (Ca and Mg), and some other metallic elements (Fe, Cu, Zn, Mn), significantly affect the pyrolysis process of cellulose, hemicellulose and lignin.

There was no formation of C1–C4 compounds and no formation of levoglucosan. The percent of phenolic compounds ranged from 10.3 to 17.6%, with the highest percentage being observed for WS, which presented 57% of K_2_O (Table 4). These results emphasize the potential of the catalytic effect of Eom et al.^38^ demonstrated that K had a catalytic effect on cellulose pyrolysis, in which there was suppression of levoglucosan formation and an increase in the yields of low molecular weight compounds.

It is important to highlight that in addition to K, Mg and Ca also promote secondary cracking reactions of pyrolysis products, converting long-chain compounds into lower molecular weight products^39^. The second most abundant element in the cotton residues evaluated in this work was Ca, but Mg was also detected in the samples with the exception of in the BCM sample. Phenolic compounds and alkyl-substituted cyclopentenones are derived from the thermal degradation of lignin under the influence of Cao.

Higher furan values were observed in the WS and SCM samples, which also had higher cellulose contents (Table 2). Furans are typical products of cellulose pyrolysis^40^. The presence of oxygenated compounds in the pyrolysis products makes it difficult to use bio-oil as a fuel, since they reduce the calorific value of the bio-oil, in addition to being unstable and corrosive due to the presence of highly reactive species such as aldehydes, acids and ketones^41^. However, it is a considerable source of several chemical compounds of great interest to the chemical industry. This bio-oil can undergo improvement processes (upgrading) to make its physicochemical properties viable as a fuel.

This study points out that colored cotton waste has potential for application in energy production processes via thermal conversion. These wastes, when disposed of incorrectly, can pollute the environment. So, if they are used for energy production, it brings economic and environmental benefits to the industry.

## Conclusions

The characterization of different parts (stalks and bolls) of colored cotton species (Green, Ruby, Topaz and Jade) isolated and its mixtures were evaluated, showing that their composition and energetic potential is very similar, eliminating the need to separate species for their application in thermochemical processes. Moreover, the mixture between bolls and stalks presented adequate characteristics to be used as an alternative energy through bio-oil obtention, such as high volatile content and considerable HHV.

The energy potential and chemical composition of the condensable pyrolysis products of different colored cotton species are similar to each other, verifying that the mixture of the parts has the potential to be used as an alternative energy source in thermochemical processes. It has adequate characteristics, such as considerable heating value, fixed carbon and high volatile content values which can be converted into bio-oil, without the need to separate the bolls and stalks. The analysis of the pyrolysis products showed a majority composition of oxygenates, which can be used as an energy carrier in other processes or be refined to obtain bioproducts such as benzene, toluene, and furans. The use of cotton waste as an energy source is an environmentally sustainable process capable of inserting by-products as an energetic source into the market, contributing to waste management and minimizing costs.

### Supplementary Information

Supplementary Tables.

## Data Availability

All data generated and analysed during the current study are available from the corresponding author on reasonable request.
