# Supplementary material for: Colored cotton crop wastes valorization through pyrolysis: a study of energetic characterization and analytical Py-GC/MS
Source: Sci Rep. 2024 Apr 23;14:9359. doi: 10.1038/s41598-024-60019-4 (PMC11039636; doi:10.1038/s41598-024-60019-4)
Supplement: Supplementary file 1 — Supplementary Tables. [file 41598_2024_60019_MOESM1_ESM.docx]

**Supporting Information**

**Colored cotton crop wastes valorization through pyrolysis: a study of energetic characterization and analytical Py-GC/MS**

*Janduir E. Silva^a^, Joemil O. D. Junior^b^, Guilherme Q. Calixto^a^, Dulce M. A. Melo^c^, Marcus A.  F. Melo^ab^, Vital C. B. Júnior^d^, Bruna M. E. Chagas^a^, Everaldo P. Medeiros^e^, Renata M Braga^a,d^

**S1 Table - Descriptive statistics for pure samples.**

| **Variables** | **Standard Deviation** | **Median** | **Average** | **Confidence Interval of The Difference (95%)** | |
| --- | --- | --- | --- | --- | --- |
|  |  |  |  | Lower | Higher |
| Moisture (%) | 0.84 | 7.30 | 7.460 | 6.85 | 8.06 |
| Volatile (%) | 2.44 | 73.15 | 72.11 | 70.35 | 73.86 |
| Ash (%) | 3.36 | 8.22 | 8.34 | 5.93 | 10.75 |
| Fixed Carbon (%) | 2.56 | 12.85 | 12.08 | 10.24 | 13.91 |
| HHV (kJ/kg) | 0.75 | 16.95 | 17.07 | 16.53 | 17.60 |

**S2 Table - Descriptive statistics for mixtures.**

| **Variables** | **Standard Deviation** | **Median** | **Average** | **Confidence Interval of the Difference (95%)** | |
| --- | --- | --- | --- | --- | --- |
|  |  |  |  | **Lower** | **Higher** |
| **Moisture (%)** | 1.05 | 8.5 | 8.62 | 7.31 | 9.93 |
| **Volatile (%)** | 3.48 | 70.2 | 70.68 | 66.34 | 75.01 |
| **Ash (%)** | 3.44 | 8.2 | 8.62 | 4.34 | 12.89 |
| **Fixed Carbon (%)** | 1.48 | 12.1 | 12.08 | 10.23 | 13.92 |
| **HHV (MJ/kg)** | 0.89 | 16.5 | 16.14 | 15.03 | 17.24 |
